# Examination of the Effect of Dimethyl Trisulfide in Acute Stress Mouse Model with the Potential Involvement of the TRPA1 Ion Channel

**DOI:** 10.3390/ijms25147701

**Published:** 2024-07-13

**Authors:** Kitti Göntér, Ágnes Dombi, Viktória Kormos, Erika Pintér, Gábor Pozsgai

**Affiliations:** 1 Department of Pharmacology and Pharmacotherapy, Medical School, University of Pécs, H-7624 Pécs, Hungary; gonter.kitti@pte.hu (K.G.); viktoria.kormos@aok.pte.hu (V.K.); erika.pinter@aok.pte.hu (E.P.); 2 Department of Pharmacology, Faculty of Pharmacy, University of Pécs, H-7624 Pécs, Hungary; agnes.dombi@pte.hu

**Keywords:** TRPA1, ion channels, dimethyl trisulfide, acute stress, endocannabinoids, substance P

## Abstract

Polysulfides are endogenously produced in mammals and generally associated with protective functions. Our aim was to investigate the effect of dimethyl trisulfide (DMTS) in a mouse model of acute stress. DMTS activates transient receptor potential ankyrin 1 (TRPA1) channels and leads to neuropeptide release, potentially that of substance P (SP). We hypothesize that DMTS might inhibit the degrading enzymes of endocannabinoids, so this system was also investigated as another possible pathway for mediating the effects of DMTS. *Trpa1* gene wild-type (WT) and knockout (KO) mice were used to confirm the role of the TRPA1 ion channel in mediating the effects of DMTS. C57BL/6J, *NK1* gene KO, and *Tac1* gene KO mice were used to evaluate the effect of DMTS on the release and expression of SP. Some C57BL/6J animals were treated with AM251, an inhibitor of the cannabinoid CB1 receptor, to elucidate the role of the endocannabinoid system in these processes. Open field test (OFT) and forced swim test (FST) were performed in each mouse strain. A tail suspension test (TST) was performed in *Trpa1* WT and KO animals. C-FOS immunohistochemistry was carried out on *Trpa1* WT and KO animals. The DMTS treatment increased the number of highly active periods and decreased immobility time in the FST in WT animals, but had no effect on the *Trpa1* KO mice. The DMTS administration induced neuronal activation in the *Trpa1* WT mice in the stress-related brain areas, such as the locus coeruleus, dorsal raphe nucleus, lateral septum, paraventricular nucleus of the thalamus, and paraventricular nucleus of the hypothalamus. DMTS may have a potential role in the regulation of stress-related processes, and the TRPA1 ion channel may also be involved in mediating the effects of DMTS. DMTS can be an ideal candidate for further study as a potential remedy for stress-related disorders.

## 1. Introduction

Stress is the universal response of an organism to any real or perceived external or internal source of threat that compromises the integrity and balance of the organism and forces the individual to adapt in order to survive [1]. Among stress adaptation disorders, depression and anxiety are the most common psychiatric diseases. There is a growing number of drugs available for the treatment of anxiety and depression, but in a significant proportion of patients, these do not lead to improvement, even after weeks of continuous treatment [2], which raises the importance of research on this topic. Polysulfides are endogenously produced in mammals and generally associated with protective functions, e.g., their antioxidant, neuroprotective, analgesic, and anti-inflammatory effects have been described [3,4,5]. Although the role of sulfide in the central nervous system is being actively researched, not much is known about its effects on stress-related behavior. The biological effects of sulfide are mediated either by reaction with heme-bound iron or by the modification of the sulfhydryl groups of cysteine amino acids in proteins [6]. The latter process actually involves the formation of polysulfides [7]. Polysulfides have been detected in the central nervous system of mice and humans [3,8]. The beneficial effects of sulfides have been reported previously during acute stress. Sodium hydrogen sulfide treatment attenuated the effect of acute stress in the forced swim test (FST) and the tail suspension test (TST) [9,10].

Inorganic polysulfide (POLY) is not an ideal candidate for drug development due to its reactivity and short half-life. Dimethyl trisulfide (DMTS), found in garlic, is more stable and has favorable pharmacokinetics [11]. The effects of DMTS on acute stress have not yet been investigated. Several effects of DMTS (e.g., analgesic and anti-inflammatory effects) are mediated by transient receptor potential ankyrin 1 (TRPA1) ion channel activation [4,12].

The TRPA1 ion channel is found in astrocytes, where it is activated by sodium polysulfide [13]. The presence of *Trpa1* in the Edinger–Westphal nucleus was confirmed by RNAscope in situ hybridization (ISH) [14], which is a highly sensitive method (single molecule detection) to visualize the mRNA expression. Moreover, its functional activity was also proved using a selective, specific, and potent agonist (JT010) by patch clamp electrophysiology in acute EW slices [15]. The locus coeruleus (LC) area was also examined and no *Trpa1* was found there by RNAscope ISH [16]. The paraventricular nucleus of the hypothalamus (PVN) and the dorsal raphe nucleus (DR) were investigated for *Trpa1* by Milicic and co-workers from our institution. They proved the low copy expression pattern of *Trpa1* in both brain areas by RNAscope ISH. The lateral septum (LS) and the paraventricular nucleus of the thalamus (PVT) have not been reported yet to express *Trpa1*.

Our previous results prove that the human TRPA1 channel expressed by the Chinese hamster ovary (CHO) cell line is activated by both POLY and DMTS [4,12,17,18]. TRPA1 expression is elevated in the dorsal root ganglion of animals exposed to acute stress [19]. In our previous experiments, DMTS was shown to greatly reduce motor activity and respiration in mice, depressing the central nervous system, mediated by the TRPA1 channel [4]. Given that central nervous system inhibitors/depressants (e.g., benzodiazepines and barbiturates) tend to reduce the levels of anxiety, we hypothesized that an appropriately chosen dose of DMTS may have an anxiolytic effect on acute stress-related behavior.

In addition to sensory neurons, substance P (SP) is also localized in the central nervous system [20]. SP and its neurokinin1 (NK1) receptors are well-known stress mediators. Neurons in some limbic structures express SP and NK1 receptors. Stress can alter SP release or receptor internalization in these brain areas. The tachykinin system has also been implicated in the pathophysiology of mood disorders. Increased SP levels can be detected in the lateral septal nucleus (LS) in response to acute stress. Mice knocked out of the *Tac1* gene encoding SP are less responsive in models of acute stress [21]. Given that the intraperitoneal administration of the H_2_S donor NaHS to mice leads to a significant elevation of circulating SP levels, sulfide contributes to the release of SP in the peripheral nervous system [22]. A similar SP release might occur in the central nervous system, too.

The main components of the endocannabinoid system (ECS) are cannabinoid receptor proteins (CB1 and CB2), ligands (endocannabinoids), and proteins involved in the regulation and metabolism of endocannabinoids. Endocannabinoids mediate retrograde signaling. They are synthesized on demand and are not stored in vesicles [23]. They are produced postsynaptic and bind to presynaptic CB receptors. The primary enzymes responsible for the hydrolysis of endocannabinoids, namely anandamide and 2-AG (2-arachidonoylglycerol) are fatty acid amide hydrolase (FAAH) and monoacylglyceride lipase (MGL). These two enzymes are important pharmacological therapeutic targets [24]. FAAH and MGL contain functional disulfide bonds that might be destroyed by DMTS, potentially inhibiting enzyme activity [25].

Animal studies have provided more direct evidence for the involvement of the ECS in anxiety and depression [26,27,28]. The ECS has been shown to function in several brain regions such as the prefrontal cortex, hippocampus, amygdala, and midbrain periaqueductal grey matter that are involved in various psychiatric disorders [29]. To date, few studies have measured endocannabinoid levels in psychiatric disorders. The basal serum concentrations of anandamide (AEA) and 2-arachidonoylglycerol (2-AG) are significantly reduced in depression, suggesting a role for this system in the disorder [30,31]. Genetic deficiency or the chronic inhibition of FAAH has an anti-anxiety and anti-depressant effect [32]. The effect of polysulfides on FAAH is likely because the activity of the enzyme can be inhibited by cysteine modification [33]. As mentioned above, MGL is also found in the central nervous system and is an important component of the ECS. Its inhibition attenuates the effects of acute stress and depression [34]. 

In summary, scientific data suggest that DMTS can (1) activate TRPA1 ion channels [4,5]; (2) release SP from neurons [22], which activates its receptor NK1; and (3) inhibit endocannabinoid-degrading enzymes [25], namely FAAH and MGL.

Our aim was to investigate the effects of DMTS in an acute stress mouse model and the potential mechanisms underlying its effects on acute stress-induced behavior. We set out to clarify the role of the endocannabinoid system in the process using the CB1 antagonist AM251. This pathway has not yet been explored in detail, so the data obtained may help to understand the possible interactions between the two conserved systems (sulfide signaling and ECS). Given the lack of data on TRPA1 expression in the LS and PVT, we also aimed to investigate *Trpa1* expression in these brain areas using RNAscope ISH.

To test our hypotheses, we used *Trpa1* WT, KO, C57BL/6J, *NK1* KO, and *Tac1* KO strains and performed open field test (OFT), FST, TST, c-Fos immunohistochemistry, and RNAscope ISH.

## 2. Results

### 2.1. Finding the Suitable Dose of DMTS via Open Field Test

**Figure 1:**
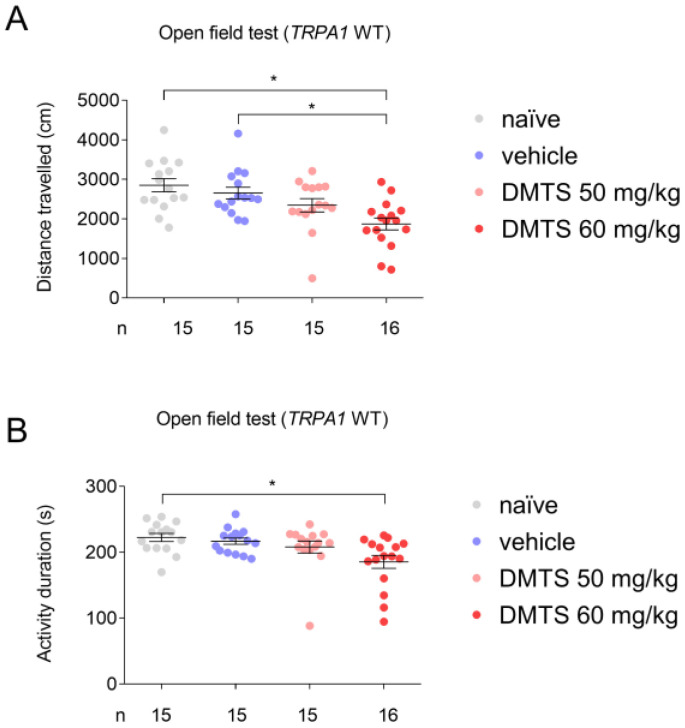
Distance travelled (**A**) and activity duration (time spent moving; (**B**)) in different treatment groups of the *Trpa1* WT mice in the open field test. Dimethyl trisulfide (DMTS) at a 60 mg/kg dose reduced, but at a 50 mg/kg dose did not affect either the distance traveled or activity duration compared to the untreated and vehicle-treated groups. One-way ANOVA and Fisher post hoc test, * *p* < 0.05 vs. the indicated group.

The time spent moving was measured in seconds and the distance covered in centimeters. DMTS at a dose of 60 mg/kg significantly reduced (*p* < 0.05) both the time spent moving and the distance traveled in the observed time frame compared to the untreated group in the Trpa1 WT strain. This negative effect was not observed at a dose of 50 mg/kg DMTS, and this dose was used in the FST and the TST. The vehicle of 50 mg/kg DMTS did not inhibit the spontaneous movement of the animals (Figure 1). 

**Figure 2:**
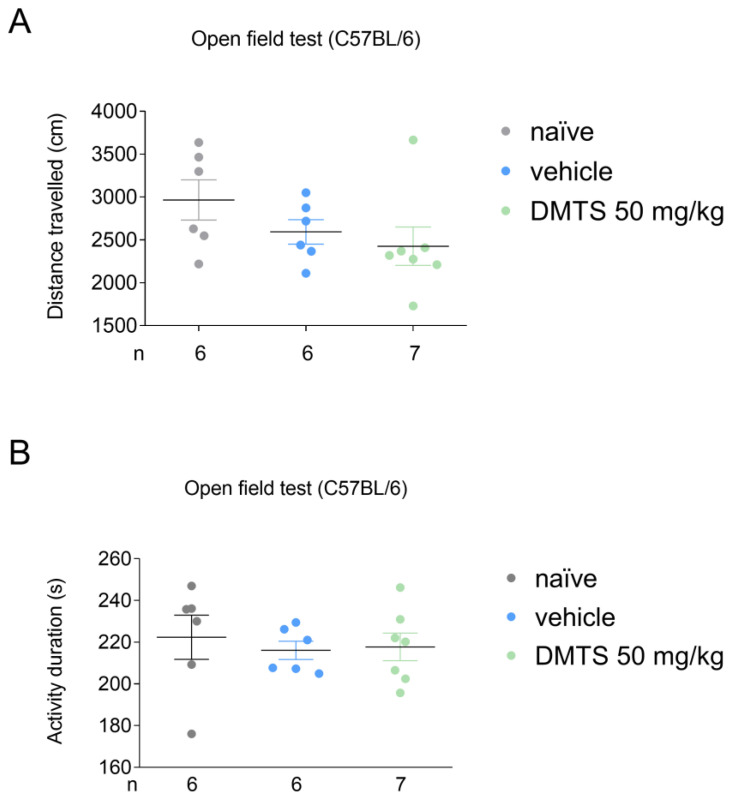
Distance travelled (**A**) and activity duration (time spent moving; (**B**)) in different treatment groups of the C57BL/6J strain in the open field test. Dimethyl trisulfide (DMTS) at a 50 mg/kg dose did not diminish either the distance traveled or activity duration in C57BL/6 mice. *n* = 6–7, one-way ANOVA and Fisher post hoc test.

In C57BL/6J mice, DMTS at a dose of 50 mg/kg and vehicle were tested compared to the untreated group. Neither DMTS (50 mg/kg) nor vehicle reduced the time spent moving and the distance traveled during the observed time period (Figure 2). Based on these results, DMTS at 50 mg/kg and the corresponding vehicle were used for the FST and the TST.

### 2.2. Behavioural Tests

#### 2.2.1. Effect of DMTS on Trpa1 WT and KO Animals in the Forced Swim Test

**Figure 3:**
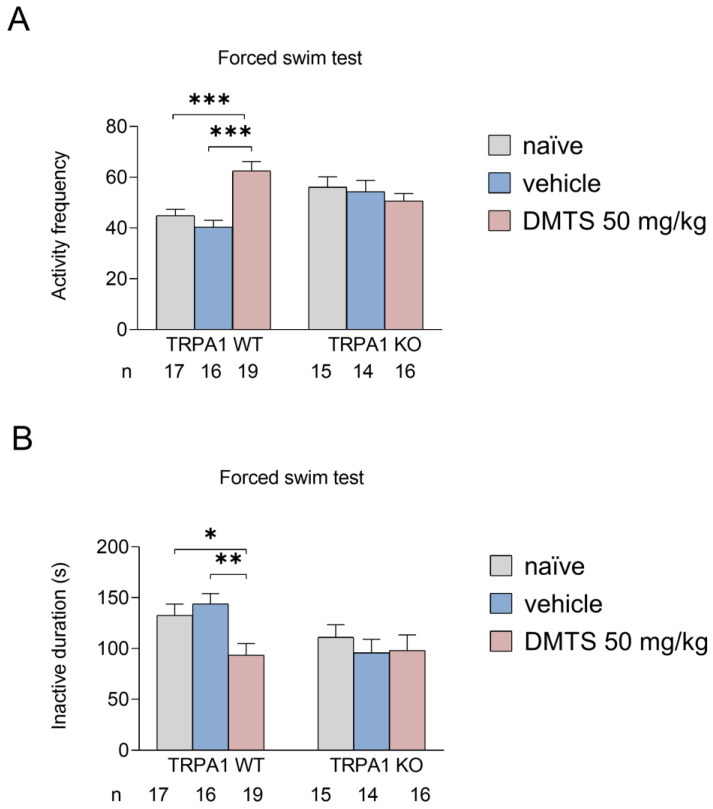
Activity frequency (**A**) and immobility time (**B**) in the forced swim test. Dimethyl trisulfide (DMTS; 50 mg/kg) increased activity frequency and decreased the time spent immobile compared to the untreated or vehicle-treated groups in the *Trpa1* WT strain. No treatment effect was observed in the *Trpa1* KO animals. Shapiro–Wilk test and Bartlett’s Chi-squared test followed by two-way ANOVA and Fisher post hoc test, *n* = 15–19, * *p* < 0.05, ** *p* < 0.01, *** *p* < 0.001 vs. indicated group.

In the behavioral test, we measured the number of times the animal entered a more active state (frequency) and the amount of time it remained inactive (seconds). The more times the animal enters an active phase and the less time it spends inactive, the better the anxiolytic effect, as the animal struggles to escape. In wild-type mice, DMTS treatment increased the frequency of active periods and decreased the inactive duration compared to both the vehicle-treated and untreated groups. Furthermore, the vehicle yielded similar results to those obtained in the untreated group. However, this protective effect of DMTS was not observed in KO animals. In the KO mice, the DMTS treatment did not increase the frequency of active periods and did not reduce the time spent inactive compared to both the vehicle-treated and untreated groups (Figure 3). 

#### 2.2.2. Effect of DMTS on Trpa1 WT and KO Animals in the Tail Suspension Test 

**Figure 4:**
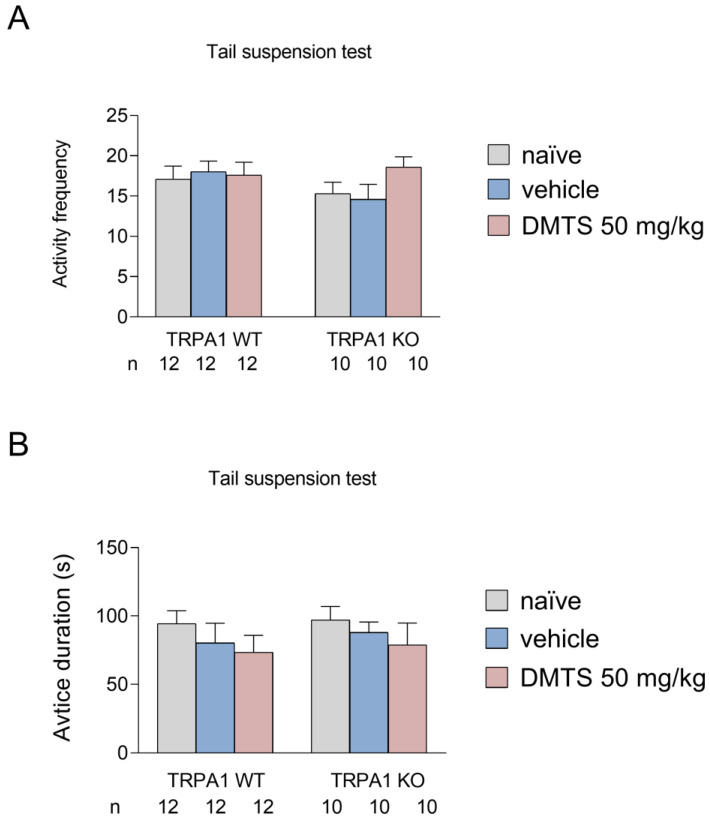
Activity frequency (**A**) and time spent immobile (**B**) in different treatment groups of *Trpa1* WT and KO mice in the tail suspension test. There was a significant effect of treatment neither on activity frequency nor on active duration in either group. Shapiro–Wilk test and Bartlett’s Chi-squared test followed by two-way ANOVA and Fisher post hoc test. *n* = 10–12. DMTS: dimethyl trisulfide.

As with the FST, we also looked at the frequency of active periods and the time spent immobile. The more often the animal engages in active movement or escape and the less time it is immobile, the better the DMTS is in reducing stress-related behavior. The graphs show the same treatment groups as shown in the previous section: untreated, vehicle-treated, and DMTS-treated (50 mg/kg). We found that DMTS did not significantly affect behavior in any strain. Although a positive trend in inactivity was observed in response to DMTS, there was no significant difference between treatment groups, and a similar trend was observed in the wild-type and KO animals (Figure 4). 

#### 2.2.3. Exploring the Involvement of Substance P in Mediating the Effect of DMTS

**Figure 5:**
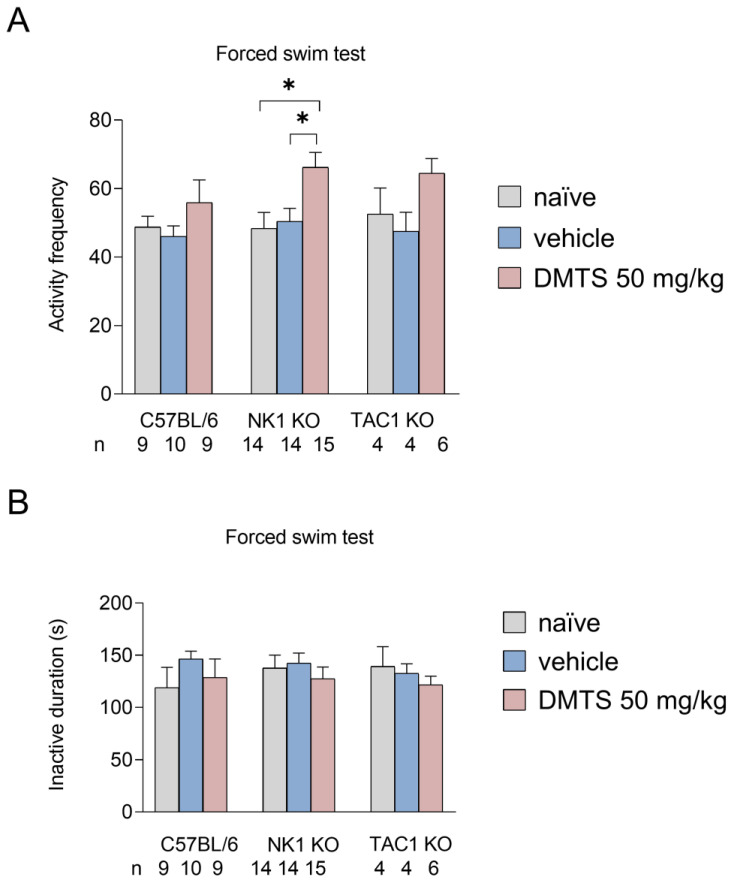
Number of active periods (**A**) and immobilization time (**B**) in different treatment groups of the C57BL/6, NK1 KO, and Tac1 KO strains in the forced swim test. The group treated with dimethyl trisulfide (DMTS) had a higher number of active periods compared to the other groups. Inactive duration was not affected by treatment in any group. Shapiro–Wilk test and Bartlett’s Chi-squared test followed by two-way ANOVA and Fisher post hoc test, *n* = 4–15, * *p* < 0.05 vs. indicated group.

We examined the time spent immobile in seconds and activity frequency in the FST. No significant differences were observed between treatment groups in the time spent inactive for either NK1 wild-type or transgenic animals. In terms of the frequency of active periods in the NK1 KO animals, DMTS increased the number of active periods of the animals compared to both the untreated and vehicle-treated groups (*p* < 0.05). A similar trend was observed in the C57BL/6J and Tac1 KO animals, but the difference was not statistically significant (Figure 5).

#### 2.2.4. Investigation of the Contribution of the Endocannabinoid System in Mediating the Effects of DMTS

**Figure 6:**
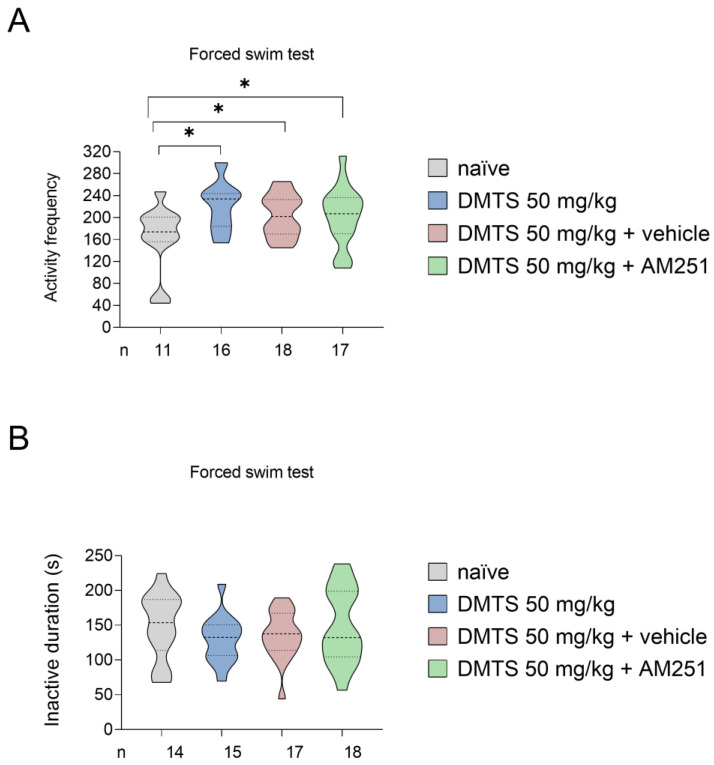
Activity frequency (**A**) and time spent immobile (**B**) in different treatment groups of the C57BL/6J strain in the forced swim test. Compared to the untreated group, all three treatments increased active frequency but had no effect on inactive duration. Shapiro–Wilk test and Bartlett’s Chi-squared test followed by one-way ANOVA and Fisher post hoc test, *n* = 11–18, * *p* < 0.05 vs. indicated group. DMTS: dimethyl trisulfide.

We also assessed the time spent immobile and the frequency of active periods in FST in the C57BL/6J mice. The DMTS treatment significantly increased the activity frequency of mice compared to the untreated group (*p* < 0.05). DMTS + AM251 co-administration significantly increased the activity frequency compared to the untreated group (*p* < 0.05), but not in relation to the DMTS-treated group. Furthermore, there was no difference between the effects of DMTS treatment, DMTS + AM251 co-treatment, and DMTS + vehicle co-treatment. The duration of inactivity was not affected by the DMTS treatment or DMTS + AM251 treatment (Figure 6).

### 2.3. Immunohistochemistry 

**Figure 7:**
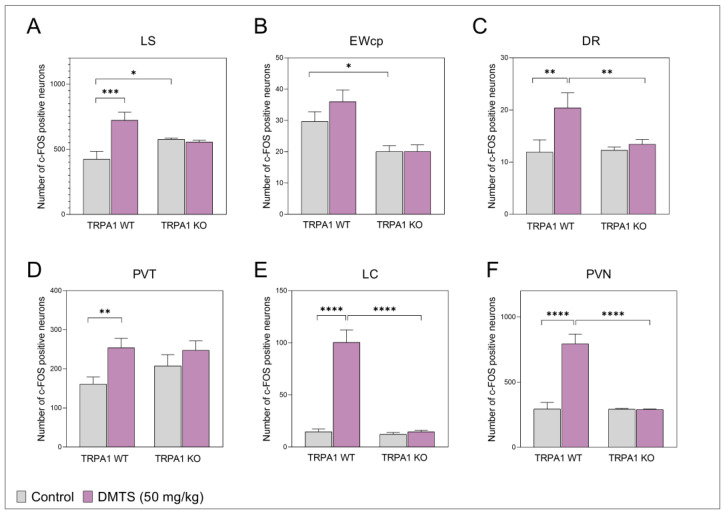
c-Fos immunoreactivity in different brain areas in vehicle-treated and dimethyl trisulfide (DMTS)-treated animals in the *Trpa1* WT and KO mouse strains. Compared to the untreated groups, the DMTS (50 mg/kg) treatment significantly increased c-Fos immunoreactivity in all the tested brain areas, except in the centrally projecting Edinger–Westphal nucleus (EWcp, (**B**)). PVN: paraventricular nucleus of the hypothalamus (**F**); LC: locus coeruleus (**E**); PVT: paraventricular nucleus of the thalamus (**D**); DR: dorsal raphe nucleus (**C**); LS: lateral septum (**A**). Shapiro–Wilk test and Bartlett’s Chi-squared test followed by one-way ANOVA and Fisher post hoc test, *n* = 10–15, * *p* < 0.05, ** *p* < 0.01, *** *p* < 0.001, **** *p* < 0.0001 vs. indicated group. Control = vehicle-treated; DMTS = dimethyl trisulfide.

**Figure 8:**
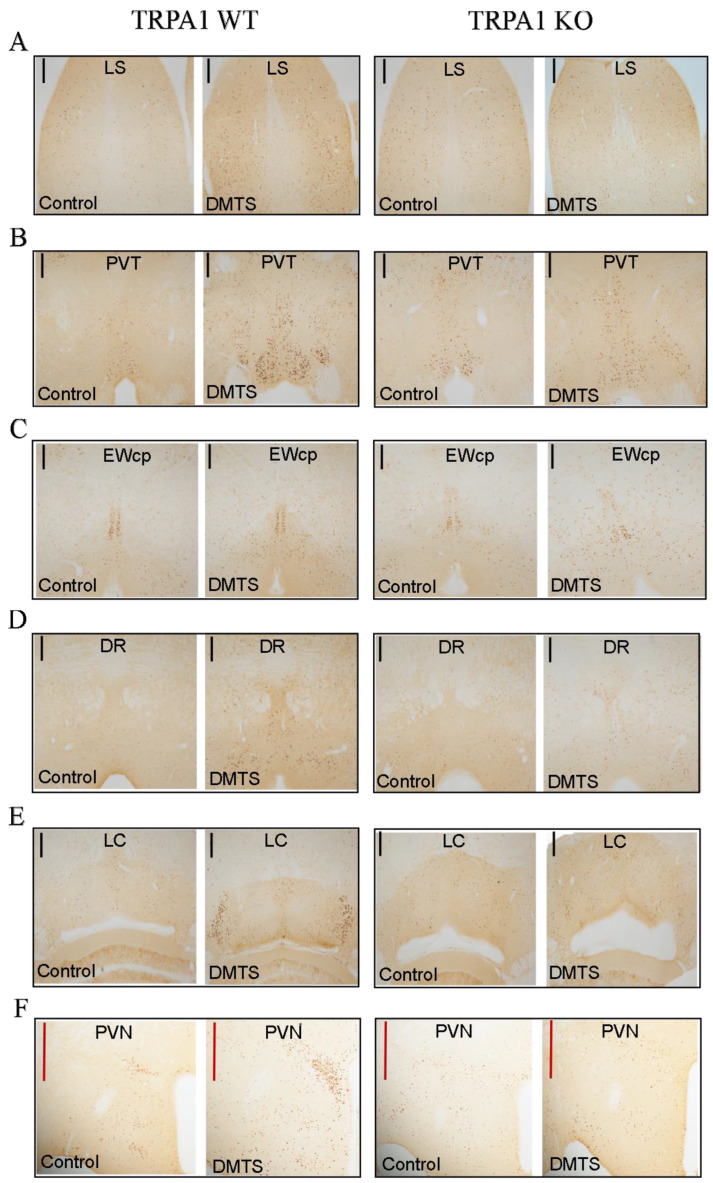
Representative images used for the calculation of the density of c-Fos activation in the brain areas of the *Trpa1* WT and KO animals. Immunohistochemistry was visualized with 3,3′-diaminobenzidine. The images were taken with a 10× objective. Apart from the centrally projecting Edinger–Westphal nucleus (EWcp), significantly stronger c-Fos activity was found in all the brain areas examined after the dimethyl trisulfide (DMTS) treatment. LS (lateral septum, (**A**)), PVT (paraventricular nucleus of the thalamus, (**B**); EWcp (**C**)), DR (dorsal raphe nucleus, (**D**)), LC (locus coeruleus, (**E**)), PVN (paraventricular nucleus of the hypothalamus, (**F**)). Control = vehicle-treated. Black line: scale bar 200 μm; red line: scale bar 500 μm.

c-Fos immunoreactivity was examined in the stress-related brain areas comparing brain samples from the vehicle-treated and DMTS-treated animals to reveal if the DMTS treatment has an effect on acute neuronal activity in these brain areas (n = 10–15). DMTS or vehicle were administered to the *Trpa1* WT and KO animals to identify areas activated by the substance (Figure 7 and Figure 8). The number of c-Fos positive neurons in the LS (Figure 7A) of the *Trpa1* WT mice increased upon treatment (main effect of treatment; *p* = 0.0027), compared to the WT control animals (Table 1). Interestingly, the c-Fos immunoreactivity of *Trpa1* KO, vehicle-treated animals was found to be slightly higher than that of their WT counterparts, but the DMTS treatment did not increase the neuronal activity in comparison to that observed in the *Trpa1* WT strain (treatment × genotype interaction; *p* = 0.0006). There was a significant increase in the c-Fos immunoreactivity of the DR of the *Trpa1* WT mice (Figure 7C) compared to the WT control group (main effect of treatment; *p* = 0.0145). There was no difference in the c-Fos immunoreactivity of the DR brain area between the two strains, but in the *Trpa1* KO strain, the DMTS treatment did not increase neuronal activity compared to the KO control group. No interaction was observed (Table 1). In the PVT area (Figure 7D), the DMTS treatment resulted in nearly one and a half times more c-Fos positivity in comparison to the control group in the *Trpa1* WT strain (main effect of treatment; *p* = 0.0081). No significant difference was found between the basal neuronal activity of the two strains, although the tendency shows that the KO animals have higher immunoreactivity. There was a tendency for the DMTS treatment to increase neuronal activity in KO animals, but this was not statistically significant. No interaction was observed (Table 1). In the LC (Figure 7E), we observed the highest c-Fos immunoreactivity boost in the DMTS-treated animals (main effect of treatment; *p* < 0.0001) compared to control mice, but only in the *Trpa1* WT animals (main effect of genotype; *p* < 0.0001). In the KO animals, the number of c-Fos positive neurons in the LC brain area did not increase as a result of the DMTS treatment (treatment × genotype; *p* < 0.0001; Table 1). We also found a nearly triple increase in c-Fos immunoreactivity in the PVN area (Figure 7F) of the *Trpa1* WT animals after the DMTS treatment compared to the vehicle-treated group (main effect of treatment; *p* < 0.0001). Similar to the LC brain area, the KO animals did not show enhanced immunoreactivity after the DMTS treatment in the PVN (main effect of genotype; *p* < 0.0001), thus there was a significant difference in the neuronal activity of the DMTS-treated animals between the two strains (treatment × genotype interaction; *p* < 0.0001; Table 1). In the EWcp (Figure 7B), we observed a mild increase in c-Fos activity after the DMTS treatment in the *Trpa1* WT animals, but the number of c-Fos positive neurons was not statistically significant compared to the WT control group (no treatment effect; *p* = 0.2676). In addition, a significant difference was found between the two strains in the number of c-Fos positive neurons in the control animals (main effect of genotype; *p* < 0.0001). The c-Fos immunoreactivity of the EWcp of the KO animals was slightly but significantly lower than that of their WT counterparts. This reactivity was not altered by the DMTS treatment. No interaction was observed (Table 1).

### 2.4. RNAscope In Situ hybridization

**Figure 9:**
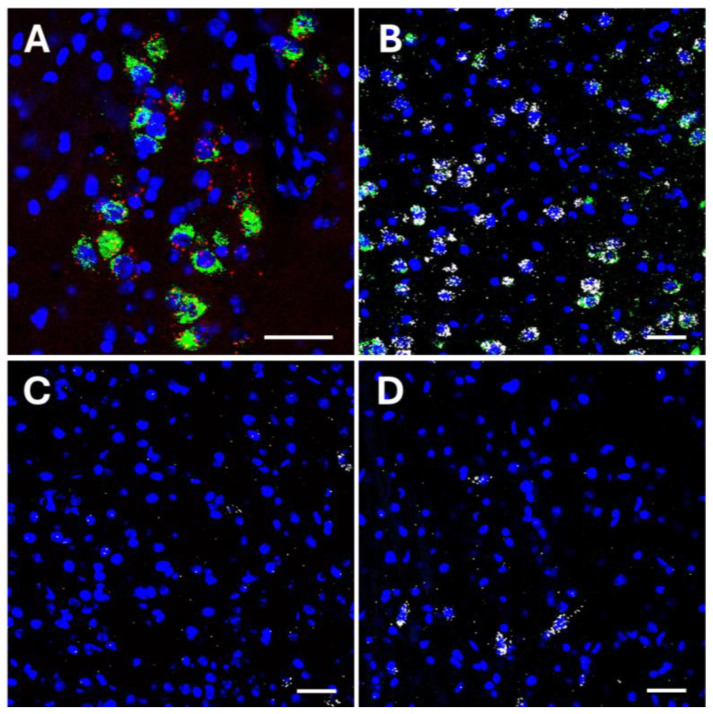
Transient receptor potential ankyrin 1 (*Trpa1*) mRNA expression. Representative fluorescence images showing the *Trpa1* mRNA transcripts (red) co-expressed with urocortin 1 (UCN1) peptide (green) in the mouse centrally projecting Edinger–Westphal nucleus (EWcp; (**A**)). Representative fluorescence images showing the *Trpa1* (red), vesicular glutamate transporter 1 (*Vglut1*, green), and vesicular glutamate transporter 2 (*Vglut2*, white) mRNA transcripts in the mouse paraventricular thalamic nucleus (PVT; (**B**)), ventral (**C**), and dorsal (**D**) part of the lateral septum (LS). Note that *Trpa1* co-localizes with UCN1 in the EWcp; however, there is no *Trpa1* in the PVT and LS. Nuclear counterstaining was performed with 4′,6-diamidino-2-phenylindole (DAPI, blue). Scale bar: 20 µm.

To explore the presence of the *Trpa1* mRNA expression in the LS and PVT, we performed RNAscope ISH for *Trpa1* in the C57BL/6J mice in these brain areas. As a positive control, the EWcp was also examined. The *Trpa1* mRNA expression was not found in either the dorsal or ventral part of the LS or in the PVT (Figure 9).

## 3. Discussion

Since the effect of DMTS on the general activity of the animals has not been studied before, the first aim of our research was to determine the appropriate dose of it that does not limit the spontaneous movement and locomotor activity of the animals. For this purpose, we exposed *Trpa1* wild-type animals to an OFT. Our results showed that DMTS at a dose of 50 mg/kg is suitable for further experiments and does not negatively affect the locomotor activity of the animals.

We then investigated the effects of a single dose of DMTS in models of acute stress, namely the FST and TST. In the FST, we found that in the *Trpa1* WT strain, the DMTS treatment increased the frequency of active periods and decreased the time spent immobile. Our findings are in the same line as those of Chen and co-workers, who tested an H_2_S donor compound in FST, and found that the treatment reduced the inactivity of the animals to the extent that we found in our study [9]. However, Chen and colleagues used chronic stress to induce depression-like behavior and also administered the treatment chronically to the animals for 7 days and no organic polysulfide was administered. Given that the effects of sulfide are mediated by polysulfides according to the literature [6,13,35], testing DMTS might be more relevant. We did not observe the stress-relieving effect in the *Trpa1* KO animals, suggesting that the TRPA1 ion channel is involved in mediating the effects of DMTS on stress-induced behavior. The role of TRPA1 itself in stress-related processes is well established. A recently published study by Kormos et al. shows that the *Trpa1* gene-knockout animals responded differently to the chronic variable mild stress model of depression than the wild-type ones. In addition, their research was the first to identify the presence of TRPA1 in the EWcp nucleus in mice and in humans, which is known to play an important role in anxiety and mood regulation via its urocortinergic neurons [14]. The research team also demonstrated the functional activity of the channel in the EWcp nucleus [36]. They have also investigated the role of the EWcp nucleus/TRPA1 ion channel in a mouse model of post-traumatic stress disorder (PTSD). Their results show that TRPA1 ion channel mRNA expression is decreased in the PTSD model, with a simultaneous increase in neuronal UCN1 peptide content in EWcp. This proposes the involvement of the cation channel in stress (mal)adaptation contributing to the pathomechanism of depression and PTSD [36].

In the C57BL/6J mice, we expected the same protective effect of DMTS. In the C57BL/6J wild-type, *NK1* KO receptor-deficient, and *Tac1* KO gene-deficient strains, we observed a similar trend: although the DMTS treatment reduced the time spent immobile and increased the frequency of active periods, there was no significant difference between the wild-type and gene-deficient animals, nor between the different treatment groups of each strain. The exception was the *NK1* KO strain, where an increase in the frequency of active periods was observed in response to DMTS in the acute stress models.

Taking into account the fact that we used a global knockout strain in this study, we cannot exclude the possibility that the loss of functional TRPA1 channels outside the central nervous system may have contributed to the observed behavioral alterations.

In the TST, no statistically significant difference was found between the treatment groups of any mouse strains, although the positive effect of DMTS on the activity of the mice presented as a trend in the results. The fact that we detected no genotype-related difference or effect of DMTS in the TST further supports the concept that effects might be test-specific [14]. In fact, it has been suggested that although the FST and TST produce similar results in anti-depressant-like activity studies, the mechanisms of drug response in the two tests may be different. These results, in agreement with this study, suggest that different stressors (FST/TST) affect locomotor activity differently [37].

Although we hypothesized the release of SP in response to DMTS and its role in mediating the effect of DMTS [22], we did not observe any behavioral differences in the *NK1* gene knockout animals (receptors for SP). It is worth noting that the time spent immobile in the TST was lower in the gene knockout animals, and further decreased in the DMTS-treated animals suggesting a stress-relieving effect of SP deficiency (a known stress mediator [19]) and a contribution of DMTS to this effect. However, the difference was not statistically significant. In the FST, the number of active periods in the *NK1* gene knockout animals was increased in the DMTS-treated animals, indicating that the absence of the receptor, and thus the inability of SP to act, contributes to the positive effect of DMTS. It would be worthwhile to test the relationship between DMTS and SP in another experimental setting.

Interestingly, the DMTS treatment and the combination of DMTS + AM251 increased the number of active periods compared to the näive group in the FST. However, there was no significant difference between the two treatment groups. Considering that DMTS was able to exert its effect even when administered with a CB1 receptor antagonist, it is assumed that the ECS is not involved in our model of acute stress. The role of the ECS might be worth investigating in a chronic stress model.

The *c-Fos* gene product (c-Fos protein) is a commonly used and accepted acute neuronal activation marker in neuroscience. Brain samples from animals perfused 1 h after the DMTS treatment were subjected to c-Fos immunohistochemistry. The DMTS treatment caused a significant increase in c-Fos expression in almost all the examined brain areas of the *Trpa1* WT mice. The magnitude of this increase was brain area-specific: in the LC, we observed a nearly tenfold increase in c-Fos expression, whereas in the DR and PVT areas, it was approximately 2.5-fold. Interestingly, the EWcp nucleus did not respond to the DMTS treatment, despite the fact that this brain area contains the highest expression of *Trpa1* mRNA of all the brain areas studied. We also performed RNAscope ISH to detect the *Trpa1* expression in LS and PVT, as there was no previously available data on this. We have decided to use the RNAscope method because the immunohistochemical staining of TRPA1 is challenging due to the lack of sufficiently specific commercially available antibodies [38,39,40]. Our findings suggest that the LS and PVT areas do not express *Trpa1*. In summary, based on the literature and our own observations, the *Trpa1* mRNA transcript is only detected in EWcp, DR, and PVN. It appears that both the c-Fos expression and the impact of the DMTS treatment are somehow linked to the presence of TRPA1. The neurons of the LC receive a wide variety of afferent inputs from several sources. The LC also receives excitatory inputs from the C1 area of the rostral ventrolateral medulla and is strongly interconnected with the DR. In addition, the LC contains noradrenaline-synthesizing neurons that send diffuse projections throughout the central nervous system. This system plays a significant role in responses to acute stress. Regarding EWcp, it is worth noting that the lower basal level of neuronal activation in the gene KO animals is not affected by the DMTS treatment, similar to the WT counterparts. The absence of *Trpa1* is thought to contribute to the lower baseline activation, as this brain area expresses the ion channel. However, in this brain area, the DMTS treatment no longer enhances the inherently higher neuronal activation in the WT mice. Additionally, DR and PVN are also thought to express *Trpa1* (unpublished data, manuscript in preparation by Viktória Kormos, 2024, Department of Pharmacology and Pharmacotherapy, Medical School, University of Pécs, Pécs, Hungary) so the lack of this channel may explain why these brain areas in the KO animals did not exhibit neuronal activation from the DMTS treatment, unlike their WT counterparts. All this provides evidence that the TRPA1 ion channel may play a role in mediating the effects of DMTS in brain areas that are important for stress regulation.

Although PVT does not express the ion channel, we noticed a difference in the enhancement of neuronal activation due to the DMTS treatment in KO animals. While it tended to enhance activation, it did not significantly increase activation in KO animals, in contrast to their WT counterparts. Anatomical studies have shown that PVT receives projections from several brainstem regions. The PVT receives robust input from the hypothalamus, including a dense innervation of neuropeptide-containing neurons [41]. The prefrontal cortical areas, including the infralimbic, prelimbic, and insular cortices, are a major source of the PVT input. The dorsomedial nucleus of the hypothalamus, the periaqueductal gray matter, and the lateral parabrachial nucleus are also major input sources for PVT. In addition to receiving strong projections from these areas, the PVT also receives projections from neurons distributed throughout the brainstem and hypothalamus. These more diffuse projection systems to the PVT express an impressive amount of neurochemical diversity, including fibrous projections involving all the monoamines (dopamine, noradrenaline, adrenaline, and serotonin) and a long list of neuropeptides [42]. It is important to note that some of the efferents from EWcp expressing *Trpa1* innervate PVT, which may explain the difference in the KO animals in response to the DMTS treatment [43].

Interestingly, even though *Trpa1* is not expressed in the LS area, we observed a difference between the *Trpa1* KO and WT mice after the DMTS treatment. These observations suggest that TRPA1 is involved in mediating the activation-enhancing effect of DMTS through some indirect mechanism, most likely by LS interacting with a brain area that expresses TRPA1. In addition, basal neuronal activation is also higher in the KO animals. Although LS does not express the ion channel, it is a major forebrain target of EWcp [43]. It has been shown that cocaine- and amphetamine-regulated transcript (CART)- and urocortin 1 (UCN1)-positive fibers from EWcp densely innervate LS, and it is known that UCN1 cells express TRPA1, so by indirect connection, the absence of TRPA1 in the EWcp nucleus in the KO animals may also explain the results in LS that the DMTS treatment did not increase neuronal activation in the KO animals. Although both fiber terminals are detected throughout the LS, UCN1-positive fiber terminals appear to be more concentrated in the ventrolateral part of the LS, whereas CART-positive ones are more concentrated towards the medial sections of the LS [44]. The LS has been shown to play a key role in emotional processes and stress responses. According to some studies, the LS promotes active stress-coping behavior and is involved in a hypothalamus–pituitary–adrenal (HPA) axis inhibitory mechanism that is at least in part mediated by septal 5-HT1A receptors and does not involve a glucocorticoid-mediated feedback mechanism [45].

Both serotoninergic neurons in the DR and urocortinergic cells in the EWcp contribute to the development of stress-related mood disorders and anxiety [46]. The activation of DR neurons results in anxiety-like behavior, as confirmed by c-Fos. [47] However, it is important to mention that although the TRPA1 ion channel is detected in certain cells of the DR, these are not the serotonergic cells, implying that there were different ways of mediating the effects of DMTS in this area. Furthermore, EWcp urocortinergic neurons interact with DR serotoninergic neurons; there is a back-and-forth connection between the two nuclei. The EWcp projection is sent to the DR, where there are corticotropin-releasing factor (CRF) receptors, which are affected by UCN1, and affects serotonin release, and therefore mood and anxiety [48,49]. It is worth noting that although c-Fos is a widely used and well-validated marker of acute neuronal activation, it does not provide information on neuronal inhibition, although the latter may also play an important role in the observed differences [50].

Increased basal c-Fos activity in the EWcp, which did not increase further by the treatment, may contribute to the reduced sensitivity of the HPA axis, suggesting that the positive effect of the treatment on activity during acute stress, in the FST, might be mediated through this area. Interestingly, among the brain areas tested, only the EWcp nucleus shows evidence of the TRPA1 ion channel expression in certain neurons [14,15], and no effect of DMTS was observed in the KO animals in some behavioral tests. The urocortinergic neurons from the EWcp nucleus project primarily to the DR and PVN areas, where they are involved in modulating the stress response [49,51,52], which may further explain why the DR area has the lowest increase in the c-Fos activation.

Briefly, these results suggest that it is not exclusively the TRPA1 ion channel that mediates the effect of DMTS on stress adaptation. This is confirmed by the elevated activation in the LC, LS, and PVT brain areas after the treatment, given that there is no TRPA1 ion channel in these brain areas [16]. Another explanation is the above-mentioned secondary activations from an area of the brain in which the TRPA1 ion channel is expressed. In addition, it was confirmed for almost all the brain areas that, in contrast to the WT mice, the DMTS treatment did not induce neuronal activity enhancement in the KO mice, suggesting a role for the TRPA1 ion channel in mediating the effects of DMTS in acute stress.

It is important to note that the *Trpa1* KO mice are developmental KO mice rather than conditional ones. There may be compensatory mechanisms that influence the behavioral phenotype of the mice in these tests. Currently, conditional *Trpa1* KO mice are not yet available in our lab, but we are currently working on it. For this reason, we were restricted to developmental KO animals in the study. Because the functional receptor was deleted in both the periphery and the brain, we could not exclude the possibility that other peripheral or central mechanisms, which we did not examine here, may have contributed to the behavioral phenotype of knockout mice [14,53,54].

In the future, we plan to test the effect of DMTS in the unpredictable mild stress mouse model of depression to explore other background mechanisms besides the TRPA1 ion channel in mediating the effect of DMTS.

## 4. Materials and Methods

### 4.1. Animals and Experimental Design

*Trpa1* WT and KO, C57BL/6J, *NK1* KO, and *Tac1* KO mouse strains were kept under standard conditions at the Department of Pharmacology and Pharmacotherapy, University of Pécs. The *Trpa1* WT and KO homozygous lines were bred separately after being backcrossed into the C57BL/6J mice for 8 generations. In the *Trpa1* KO mice, although the TRPA1 ion channels are present, they are non-functional. The SP receptor is absent in the *NK1* KO mice, and the *Tac1* KO animals lack the *Tac1* gene, which is responsible for encoding substance P and neurokinin A. The C57BL/6J strain is the wild type of the *NK1* KO and *Tac1* KO strains. Male, 8–12 weeks old, 25–30 g animals were used in our experiments.

In the first experiment, OFT was used to determine the appropriate intraperitoneal doses of DMTS (Sigma-Aldrich, Budapest, Hungary) and vehicle, in which the C57BL/6J and *Trpa1* WT animals were divided into three treatment groups: untreated (naïve), treated with vehicle, and treated with DMTS. A solution of 3% m/v was prepared by dissolving polysorbate 80 in physiological saline. By dissolving DMTS at a concentration of 10 mg/mL, the DMTS stock solution was obtained. The solutions were diluted further with physiological saline to produce working solutions. The solutions were administered intraperitoneally to the animals. The vehicle used in the experiments contained 1.5% m/v polysorbate 80 (Sigma-Aldrich, Budapest, Hungary).

After determining the appropriate dose of DMTS, the animals were subjected to various behavioral tests. We investigated the effect of DMTS in an acute stress model by FST and TST using the *Trpa1* WT and *Trpa1* KO animals in a similar method to OFT using the naive, vehicle, and DMTS-treated groups. The potential impact of substance P in acute stress situations was examined only by FST in the C57BL/6, *NK1* KO, and *TAC1* KO mice, also dividing the animals into the three groups mentioned above.

The role of the endocannabinoid system in the behavior during acute stress was evaluated in the C57BL/6J strain using FST. AM251 (Sigma-Aldrich, Budapest, Hungary) was prepared in DMSO (Sigma-Aldrich, Budapest, Hungary; 10 mg/mL) and in this case, the animals were divided into 4 groups: naïve, DMTS (50 mg/kg) treated, DMTS and vehicle co-treated, and DMTS and AM251 (3 mg/kg) co-treated. DMTS was administered intraperitoneally half an hour before the experiments and AM251 was administered subcutaneously into the interscapular region half an hour before the DMTS treatment to avoid drug interaction.

Finally, stress-related brain areas were analyzed in the *Trpa1* WT strain by c-Fos immunohistochemical staining using the vehicle-treated and DMTS-treated groups. A separate groups of animals were used that did not participate in the behavioral tests.

### 4.2. Behavioural Tests

#### 4.2.1. Open Field Test

**Figure 10:**
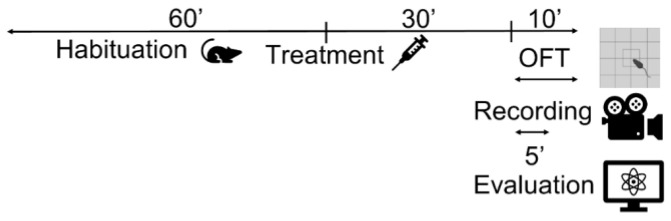
The open field test (OFT) was used to determine the appropriate dose of dimethyl trisulfide. Movement time (s) and total distance traveled (cm) were investigated. Prior to the test, we took the animals to the laboratory to habituate to the environment. Video recording lasted 10 min from which 5 min were evaluated.

The OFT was used to determine the appropriate dose of DMTS that does not reduce the locomotor activity and spontaneous movement of the animals. Although the OFT is a test used to assess locomotor activity, anxiety, and exploratory behavior in rodents, we used this method only to determine whether different doses of DMTS and their respective vehicles caused the animal to move less, the same, or more in the box [55]. The evaluation started 30 s after the appearance of the animal (this is how long we allowed them to explore the new environment) and lasted for 5 min (Figure 10). The time spent moving and the total distance covered during the observed interval were recorded using the Noldus EthoVision XT Version 15 software (Noldus Information Technology, Wageningen, The Netherlands). Doses of 50 mg/kg and 60 mg/kg of DMTS and its associated vehicle were tested. Two strains of mice were treated: male *Trpa1* WT and C57BL/6J animals.

#### 4.2.2. Forced Swim Test and Tail Suspension Test

**Figure 11:**
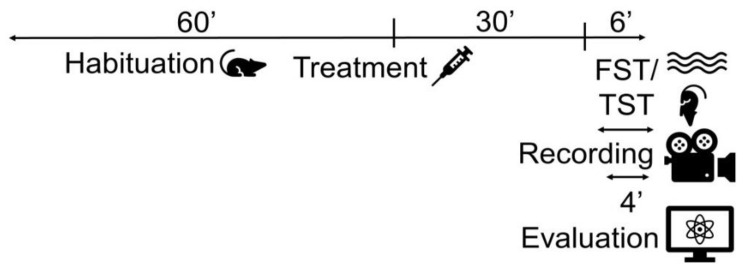
To investigate the depression-like behavior, we used the forced swim test (FST) and tail suspension test (TST). Highly active duration (s) and activity frequency parameters were utilized. The duration of immobility (s) was calculated by subtracting highly active duration from the total duration of observation.

After establishing the appropriate dose of DMTS, the experimental animals were studied in acute stress tests—FST and TST. These tests represent acute stress situations in which we can quantify depression-like behavior in animals. FST was performed with the male *Trpa1* WT and KO, C56BL/6J, *NK1* KO, and *Tac1* KO strains. TST was carried out on the *Trpa1* WT and KO strains. The animals were first exposed to the arrangement during the experiment. The treatment was performed with a single dose of DMTS or the appropriate vehicle 30 min before the behavioral tests (Figure 11).

The animals were placed in a cylindrical transparent bottle filled with 24 °C tap water to a height that they could not climb out of. The animals were placed in the water-filled bottle for 6 min each and the last 4 min were recorded to determine how much time they spent immobile and how many times they entered a high-activity phase. FST results were processed using the Noldus EthoVision XT Version 15 software. An animal was considered inactive if it floated vertically in the water and only moved its legs to keep its head above the water. Depression-like behavior is characterized by the animal giving up the struggle and floating in the water.

During TST, the mice were suspended by their tails for 6 min at a height of 50 cm. During the last 4 min, the time spent motionless and the number of times the animals entered a high-activity phase were recorded.

The experiments were carried out in a separate room so the animals could not see each other swim and the bottles were always washed after each animal. The mice were taken out of the animal facility an hour before treatment for habituation, and were acclimatized for 60–60 min on the 3 days before the experiment.

### 4.3. Perfusion and Tissue Collection

Immunohistochemistry was performed in a separate group of animals to investigate the effect of the DMTS treatment on specific brain areas in the *Trpa1* wild-type strain. Animals were anaesthetized with urethane (intraperitoneal, 2.4 g/kg), and transcardially perfused with 100 µM phosphate-buffered saline (PBS) followed by 4% m/V paraformaldehyde (in 200 µM Millonig buffer). Brain tissue was collected from each animal and postfixed in 4% m/V paraformaldehyde. Coronal sections of 30 µm thickness were made using a Leica VT1000S vibratome. The sections were stored in PBS containing 0.01% sodium azide in 4 °C. Immunohistochemistry was performed to detect the expression of the c-Fos protein in the centrally projecting Edinger–Westphal nucleus (EWcp) (Bregma −2.92 mm to −4.04 mm), dorsal raphe nucleus (DR) (Bregma −4.04 to −4.16 mm), locus coeruleus (LC) (Bregma −5.34 to −5.40 mm), lateral septum (LS) (Bregma 0.26 to −0.10 mm), paraventricular nucleus of the thalamus (PVT) (Bregma −0.22 to −0.70 mm), and paraventricular nucleus of the hypothalamus (PVN) (Bregma 0.26 mm).

#### 4.3.1. c-Fos Immunohistochemistry

The sections were washed in 0.1M PBS (pH 7.6) for 3 × 10 min. The inhibition of endogenous peroxidase activity was performed in 1% H_2_O_2_ solution in PBS for 30 min. After washing steps, the sections were treated with 0.5% Triton X-100, and aspecific binding sites were blocked by 2% normal goat serum in PBS for 30 min. The sections were incubated overnight at room temperature with rabbit polyclonal anti-c-Fos antibody in a 1:500 diluted solution (Santa Cruz Biotechnology Inc., sc-52, Santa Cruz, CA, USA) which was dissolved in PBS containing 2% normal goat serum. After washing steps, sections were incubated with a biotinylated secondary antibody (biotinylated anti-rabbit gamma globulin; Vectastain Elite ABC-HRP Kit). This was followed by incubation for one hour in an avidin–biotin complex (Vectastain Elite ABC Kit) at 20 °C. After washing in PBS, a Tris buffer containing 0.02% DAB and 0.00003% H_2_O_2_ was added to the sections. The reaction was monitored under a microscope, measured with a stopwatch, and stopped with PBS when the optimal contrast background was reached. The sections were mounted on gelatine-coated slides and dried overnight at room temperature. The slides were then dehydrated in alcohol (70%, 96%, absolute alcohol 10–10–10 min), followed by xylene treatment (2 × 20–60 min). The slides were coverslipped with DePex.

#### 4.3.2. RNAscope In Situ Hybridization

The pretreatment procedure was optimized for 30 µm-thick PFA-fixed sections [14]. The subsequent steps, including probe hybridization, signal amplification, and channel development, were carried out according to the RNAscope Multiplex Fluorescent Reagent Kit v2 user manual (ACD, Hayward, CA, USA). The mouse *Trpa1* probe (ACD; Cat. No.: 400211-C2) was visualized with cyanine 3 (Cy3) dye (1:750), vesicular glutamate transporter 1 (*Vglut1*) (ACD; Cat. No.: 416631) with Fluorescein (F) dye (1:1500), and vesicular glutamate transporter 2 (*Vglut2*) (ACD; Cat. No.: 319178-C3) with cyanine 5 (Cy5) dye (1:1500). Mouse triplex positive (ACD; Cat. No.: 320881) and triplex negative (ACD; Cat. No.: 320871) control probes were tested on the samples. The triplex positive control probes gave a well-detectable signal, while the negative control probes did not give any recognizable fluorescence in the preparations. After the RNAscope procedure, in the case of EWcp, the slides were treated with the primary antibody (rabbit anti-urocortin 1 antibody, Cat. No.: ab283503, Abcam, Cambridge, UK, 1:5000) for 24 h. After washing, the slides were incubated with a secondary antibody (Alexa Fluor 488-conjugated donkey anti-rabbit antibody, Cat. No.: 711-545-152, Jackson ImmunoResearch Laboratories, Ely, UK, 1:500) for 3 h at room temperature, then counterstained with DAPI (ACD) and cover-slipped with glycerol–PBS (1:1).

### 4.4. Evaluation Methods

#### 4.4.1. Noldus EthoVision XT 15

The Noldus EthoVision XT system was developed to monitor animal movements in a laboratory environment. Using an overhead camera, the software can track animals based on black and white contrast. This type of evaluation is reliable compared to subjective methods, provides a wide range of data, and can be consistently applied in different laboratories [56].

In the OFT, the animals were separated from the background by the software based on the black and white contrast. The evaluation starts 30 s after the animal appears and lasts for 5 min. The time spent moving and the distance traveled during this time are assessed.

In the case of the FST, once the animal was in the water, the 6 min recording started. The last 4 min were evaluated. We measured how long the animal was inactive. We also looked at how many times the animal entered a higher activity state. Activity detection is based on the changes in the outline of the animals on the frames of the recordings.

#### 4.4.2. ImageJ

After photographing the histological sections, we used ImageJ Version 1.54d software and the IHC Toolbox. By outlining the brain area to be examined and setting the appropriate thresholds, the program separates the cells from the background based on density, counts the number of cells within the area, and calculates the size of the outlined area. The IHC Toolbox extension also includes a model optimized specifically for DAB staining. The program allows manual or automatic cell counting. In our case, manual counting was not practical given the large number of cells, so we used automatic counting. We converted the RGB image to greyscale (8-bit). The calculation was based on the area of the image. In the next step, we highlighted all the structures to be counted using the threshold command. Finally, by clicking on the analyze command, the program calculated the number of structures highlighted relative to the area selected.

#### 4.4.3. Microscopy, Digital Imaging

Fluorescent-labeled sections were digitalized by an Olympus FluoView 1000 confocal microscope (Olympus, Europa, Hamburg, Germany) in analog mode by sequential scanning using 3.5 µm optical thickness and 1024 × 1024-pixel resolution. The excitation and emission spectra for the respective fluorophores were selected using built-in settings of the FluoView software (FV10-ASW; Version 0102, Olympus, Europa, Hamburg, Germany). DAPI was excited at 405 nm, Fluorescein/Alexa 488 at 488 nm, Cy3 at 550 nm, and Cy5 at 650 nm. The sections were scanned for the respective wavelengths at four channels. The digital images of the individual channels, depicting the same area, were automatically superimposed and merged.

### 4.5. Statistics

The data presented show the mean and standard error of the mean of the experimental groups. An unpaired *t*-test was used to evaluate the immunohistochemical data. The normality of the data distribution was checked by the Shapiro–Wilk test and the homogeneity of variance by Bartlett’s Chi-squared test [57]. The data obtained in the Noldus system was evaluated by the one-way analysis of variance (ANOVA) in the case of OFT. In the FST, to assess the effect of AM251, we used also a one-way ANOVA. In all the other cases, we evaluated the data by 2-way ANOVA. All the post hoc analyses were performed by the Fisher test based on first- or second-order effects in the ANOVA. Statistical evaluation was performed using GraphPad Prism 8.

## 5. Conclusions

We provide, to our knowledge, the first evidence for the effect of DMTS in the central nervous system, which may be mediated in part through the TRPA1 ion channel.

DMTS may have a specific regulatory function of acute stress-induced behavior in part through the regulation of the TRPA1 ion channel. Since similar results were obtained in the C57BL/6 wild-type, *NK1* KO receptor-deficient, and Tac1 KO gene-deficient animals, we excluded the role of SP in this process. In our ongoing research, we are investigating the effects of DMTS in a chronic unpredicted mild stress model of depression by examining the involvement of the TRPA1 ion channel and the endocannabinoid system.

DMTS may be an ideal candidate for further study as a potential substance in the regulation of stress adaptation.

### Limitations

The behavioral phenotype of our global KO mouse strain may be due to developmental compensation. We cannot exclude the possibility that other peripheral or central mechanisms, which we did not study here, may contribute to the behavioral phenotype of the KO mice, as the functional receptor was deleted both peripherally and centrally. In this study, female mice and tissue samples were not examined. Responses in female mice may be influenced by the phase of the estrous cycle, as some of the examined brain areas express the estrogen receptor ß [49,50,51,52,53].

Another limitation is related to the substance used in our research study, as DMTS has not been tested in behavioral tests before, so there is not enough data on the possible side effects of DMTS on the central nervous system and animal behavior due to the lack of previous research.

## Figures and Tables

**Table 1:** Summary of statistical analyses of c-Fos immunoreactivity of brain areas by two-way analysis of variance (ANOVA). LS = lateral septal nucleus; EWcp = centrally projecting Edinger–Westphal nucleus; DR = dorsal raphe nucleus; PVT = paraventricular nucleus of the thalamus; LC = locus coeruleus; PVN = paraventricular nucleus of the hypothalamus. Significant values are highlighted in bold.

		Main Effects		Interaction
Brain Area		Treatment	Genotype	Treatment × Genotype
LS	F_1,56_	**9.882**	0.02881	**13.05**
	*p* value	**0.0027**	0.8658	**0.0006**
EWcp	F_1,56_	1.254	**1.254**	1.254
	*p* value	0.2676	**<0.0001**	0.2676
DR	F_1,56_	**6.395**	3.058	3.736
	*p* value	**0.0145**	0.0861	0.0586
PVT	F_1,56_	**7.541**	0.6658	1.191
	*p* value	**0.0081**	0.418	0.2799
LC	F_1,56_	**48.41**	**48.35**	**43.28**
	*p* value	**<0.0001**	**<0.0001**	**<0.0001**
PVN	F_1,56_	**46.65**	**48.1**	**47.53**
	*p* value	**<0.0001**	**<0.0001**	**<0.0001**

## Data Availability

The datasets used and/or analyzed during the current study are available from the corresponding author upon reasonable request.

## Abbreviations

TRPA1: transient receptor potential ankyrin 1; DMTS, dimethyl trisulfide; DMSO, dimethyl sulfoxide; WT, wild type; KO, knockout; CHO, Chinese hamster ovary; CB1, cannabinoid receptor 1; AEA, anandamide; 2-AG, 2-arachidonoylglycerol; MGLL, monoacylglycerol lipase; FAAH, fatty-acid amide hydrolase; LC, locus coeruleus; DR, dorsal raphe nucleus; LS, lateral septum; PVN, paraventricular nucleus of the hypothalamus; PVT, paraventricular nucleus of the thalamus; EWcp, centrally projecting Edinger–Westphal nucleus; SP, substance P; NK1, neurokinin 1 receptor; FTS, forced swim test; TST, tail suspension test; OFT, open field test; MBT, marble burying test; SPT, sucrose preference test; POLY, inorganic polysulfide; Tac1, tachykinin precursor 1; PBS, phosphate-buffered saline; IHC, immunohistochemistry; DAB, 3,3′-diaminobenzidine; ANOVA, analysis of variance; PTSD, post-traumatic stress disorder; UCN1, urocortin 1; H_2_S, hydrogen sulfide; NaHS, sodium hydrosulfide; ECS, endocannabinoid system; CRF, corticotropin-releasing factor; CART, cocaine- and amphetamine-regulated transcript; ISH, in situ hybridization.
